# Curcumin activation of a bacterial mechanosensitive channel underlies its membrane permeability and adjuvant properties

**DOI:** 10.1371/journal.ppat.1010198

**Published:** 2021-12-23

**Authors:** Robin Wray, Irene Iscla, Paul Blount

**Affiliations:** Department of Physiology, UT Southwestern Medical Center, Dallas, Texas, United States of America; University of Toronto, CANADA

## Abstract

Curcumin, a natural compound isolated from the rhizome of turmeric, has been shown to have antibacterial properties. It has several physiological effects on bacteria including an apoptosis-like response involving RecA, membrane permeabilization, inhibiting septation, and it can also work synergistically with other antibiotics. The mechanism by which curcumin permeabilizes the bacterial membrane has been unclear. Most bacterial species contain a Mechanosensitive channel of large conductance, MscL, which serves the function of a biological emergency release valve; these large-pore channels open in response to membrane tension from osmotic shifts and, to avoid cell lysis, allow the release of solutes from the cytoplasm. Here we show that the MscL channel underlies the membrane permeabilization by curcumin as well as its synergistic properties with other antibiotics, by allowing access of antibiotics to the cytoplasm; MscL also appears to have an inhibitory role in septation, which is enhanced when activated by curcumin.

## Author summary

The rhizome of turmeric contains a compound, called curcumin, which has been shown to have several biological activities, including antibacterial properties. Previous studies have found that upon curcumin treatment, bacterial cells become “leaky”; their membranes permeabilized. In addition, curcumin appears to work in synergy with several other antibiotics. Finally, it has been observed that curcumin can lead to bacterial cells that can grow but not divide, thus leading to long filamentous cells with multiple copies of their DNA. But the mechanisms underlying these findings were mysterious. Here we show that curcumin targets a specific protein in the bacterial cell envelope that can open a very large pore—the largest regulated pore known in the biological world. This pore usually is only used in extreme situations as an “emergency release valve” that prevents lysis from osmotic stresses. It is the inappropriate opening of this pore by curcumin that leads to the permeation of the bacterial cell envelope, as well as the synergy with other antibiotics by allowing the antibiotics easier access to the inside of the cell, and even the inhibition of bacterial cell division.

## Introduction

Curcumin is a flavonoid polyphenol isolated from the rhizome of turmeric (*Curcuma longa*) and has been shown to have possible therapeutic potential in treatment of cancer and inflammation, and has substantial antibacterial properties [1]. It has excellent therapeutic potentials and has been found to be safe and well-tolerated up to a dose as high as 12 g/day without toxicity in clinical trials. It is thus “Generally regarded as safe” (GRAS) by the US FDA [2,3]. Curcumin has been reported to have several physiological effects in bacteria including an apoptosis-like response involving RecA in *E*. *coli* [4], membrane permeabilization [5], and inhibiting septation [6]. It can also work synergistically with other antibiotics and serve as an adjuvant [7,8,9].

Membrane depolarization, leakage and permeabilization are the most widely described mechanism of action of curcumin as an antibiotic agent [5]. This permeabilization appears to be detrimental to the cell for many bacterial species, and the resulting increased cytoplasmic entry of other antibiotics may be a major mechanism for the antibacterial synergy often observed. Although this effect has been reported for multiple bacterial species, the exact mechanism of curcumin “increased membrane permeabilization”, which is specific for bacterial membranes, remains unknown.

Bacteria contain mechanosensitive (MS) channels that serve the function of biological emergency release valves [10,11]. When the external osmotic environment acutely and rapidly decreases, these channels open due to membrane tension and release solutes from the cytoplasm to avoid cell lysis. These channels are divided into two distinct and unrelated families. The first family contains the MscL channels, which are highly conserved, found in most bacterial species and opens a huge pore that is the largest gated pore known on the order of 30Å. The second family contains MscS and MscS-related channels; this is a much more diverse channel family for which bacteria often have multiple members that appear to serve slightly different purposes. The inappropriate gating of the large-pore MscL channel can be detrimental to the cell as initially determined by genetic studies [12,13]; more recently, we have shown that known antibiotics [14,15] as well as recently discovered novel small compounds, 011A and K05, open the MscL channel [16,17,18] and can increase the potency of well-characterized antibiotics, by increasing cytoplasmic access.

It seemed that the opening of MscL’s 30Å pore would be consistent with the membrane permeabilization observed for curcumin and here we explore this possibility, as well as if it plays a role in the other reported curcumin induced effects. We have found that activation of the MscL channel indeed underlies the membrane permeabilization and adjuvant properties of curcumim, and appears to have a more indirect role in other physiological effects of this compound, including inhibiting septation.

## Results

### Curcumin inhibits growth and reduces viability in a MscL-dependent manner

**Fig 1:**
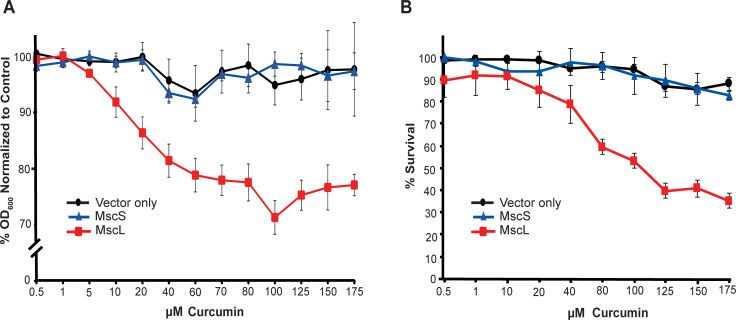
Curcumin inhibits growth and reduces viability in a MscL-dependent manner. Shown is the *E*. *coli* strain MJF455 (ΔMscL, ΔMscS) carrying vector only (black circles) or expressing MscS (blue triangles) or MscL (red squares). A) Percentage of decreased growth after treatment with curcumin (OD_600_) relative to untreated are shown. B) Reduction in viability after curcumin treatment shown as percent decrease in CFU’s relative to untreated. n = 3–5 and. error bars show standard error of the mean (SEM).

To initially determine if the decreased cell growth and viability observed from curcumin treatment was MscL dependent, we used an *E*. *coli* strain MJF455, which lacks both major mechanosensitive channels, MscL and MscS; we compared the ability of curcumin to inhibit bacterial growth in cells carrying an empty vector with those expressing MscL or MscS channels *in trans*. MscS, which also opens in response to membrane tension, was used as a control, to determine if effects are MscL specific or attributable to a generalized change in membrane tension or other biophysical properties; amphipaths that add membrane tension affect both channels [10], especially MscS [19,20]. As shown in **Fig 1A**, the growth of bacteria expressing MscL was inhibited by curcumin in a concentration dependent manner; the MscL-null control and MscS expressing bacteria were unaffected. These results could be influenced by the fluorescent nature of curcumin; we therefore also performed viability experiments independent of OD measurements. We found that when the same samples were tested for viability, only the MscL expressing cultures showed a decreased viability in a curcumin concentration dependent manner (Fig 1B). Whether it be due to curcumin’s fluorescent nature, or non-viable cells still contributing to the OD measurements, or both, it is important to note that the viability results were unambiguous and showed a more dramatic decrease upon curcumin treatment than OD measurements.

**Fig 2:**
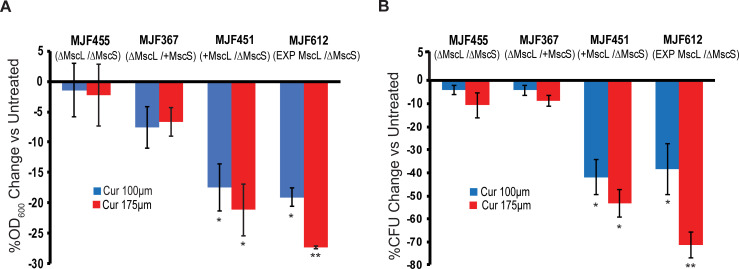
Endogenous expression levels of MscL are sufficient for curcumin antibiotic action. Shown are the *E*. *coli* Stains MJF455 (ΔMscL, ΔMscS), MJF367 (ΔMscL), MJF451 (ΔMscS), MJF612 (ΔMscL, ΔMscS, ΔMscK, ΔybdG) carrying empty plasmid (MJF455, MJF367, MJF451) or expressing MscL (MJF612) treated with curcumin at 100 μM (blue) or 175 μM (red). A) Inhibition of growth (OD_600_) in the presence of curcumin graphed as the percentage change of untreated. B) The reduction in viability of cultures shown in A as the percent change of colony forming units (CFUs) vs Untreated. Note that only when MscL is expressed either at endogenous- levels or by heterologous expression, the presence of curcumin has an effect on growth and viability. n = 3, Statistics reflect a comparison of MscL-expressing strains versus MJF455 *P < 0.05, **P < .005 as indicated, 2-tailed, homoscedastic t-test.

In the above experiment, we used an expression plasmid, pB10d with a mid-copy number per cell; the overexpression levels are only a few fold greater than endogenous expression [21]. To assure that the observed curcumin effect was not simply due to this modest over-expression, we assessed if endogenous expression levels of MscL are sufficient for curcumin action. *E*. *coli* strains derived from the parental strain FRAG1 were presented with to two different curcumin concentrations. Strains null for MscS and MscL (MJF455), MscL only (MJF367) or MscS only (MJF451) were used. To rule out the possible significant involvement of other channels and to express MscL in a “cleaner” background, these data were compared with MJF612, also a FRAG1 background, null for four bacterial mechanosensitive channels (MscL, MscS, and two other MscS-like channels), expressing MscL *in trans*. As shown in Fig 2, both growth (Fig 2A) and viability (Fig 2B) are affected by curcumin only in strains expressing MscL regardless if the expression is at endogenous levels or via our expression vector. Note that in both endogenous and *in trans* expressing MscL strains the effects of curcumin are comparable and concentration dependent.

### Curcumin increases MscL activity in native bacterial membranes as assayed by patch-clamp

**Fig 3:**
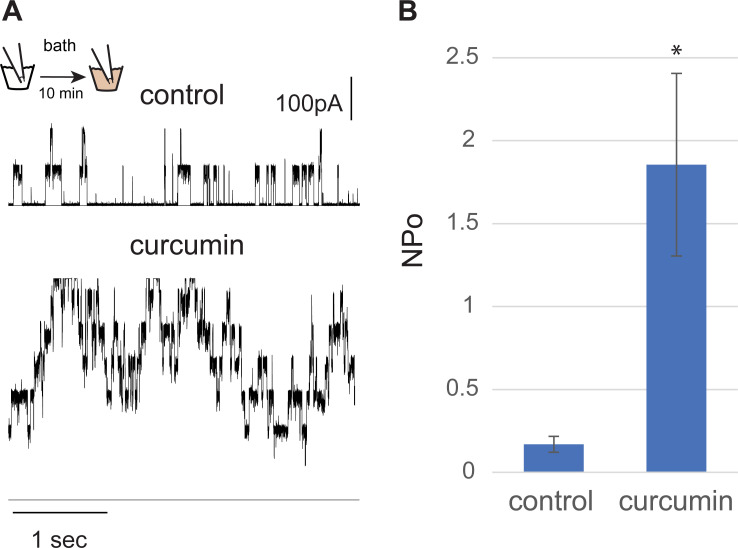
Curcumin increases MscL activity in native bacterial membranes. A) Representative traces of MscL activity in an excised patch from bacterial spheroplasts, held at a pressure of -120 mmHg, before (control) and after 10 minutes of the addition of curcumin 100 μM to the bath. B) Quantification of MscL channel activity measured as the open probability (NPo) of the channels in a patch held at the same pressure, before and after curcumin treatment. * p<0.05 Student t-test, paired, (n = 4).

To more directly assay the effects of curcumin on MscL activation, MscL channel activity was studied by patch clamp in native bacterial membranes using the giant spheroplast preparation [21]. MscL was activated by applying negative pressure to the patch, and its activity compared in the same patch before and after curcumin treatment. Representative traces of MscL channel activity are shown in Fig 3A, before (upper traces) and after 10 minutes of curcumin addition to the bath (lower traces). The robust increase in MscL activity observed in the presence of curcumin was quantified as a significant decrease in MscL pressure activation threshold, from -142.5±16 before to -109.2±24 mmHg after curcumin treatment (n = 4, p≤0.05, Student t-test paired); an increased open probability, as shown in Fig 3B was also apparent. Such an increase in MscL sensitivity is consistent with that seen for gain-of-function mutants [13] and agonists [16,17,18]. These results, combined with the *in vivo* data above, show that curcumin directly activates the MscL channel in native bacterial membranes.

### Both RecA and MscL are required for curcumin-dependent decreases in growth and viability

**Fig 4:**
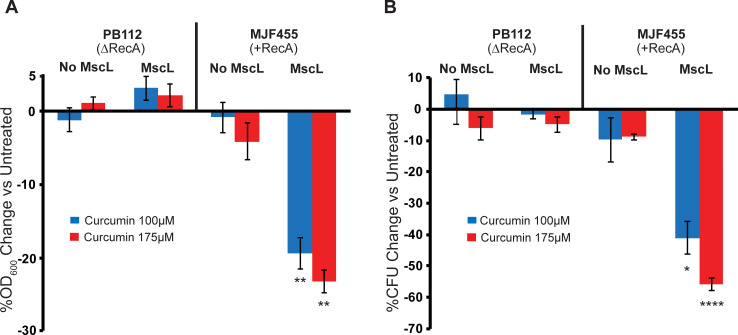
Both RecA and MscL are required for curcumin antibiotic action. Shown are the *E*. *coli* Stains PB112 (ΔrecA, ΔMscL, ΔMscS) and MJF455 (ΔMscL, ΔMscS) carrying vector only (No MscL) or expressing MscL (MscL), treated with curcumin at 100 μM (blue) or 175 μM (red). A) Inhibition of growth (OD_600_) in the presence of curcumin expressed as the percentage change of the untreated. B) The reduction in viability of cultures shown in A, expressed as the percent change of colony forming units (CFUs) vs Untreated. n = 3–5, Statistics reflect a comparison of MscL-expressing versus “no MscL” strains *P < 0.05, **P < 0.005 ****P < 0.00005 as indicated, 2 tailed, homoscedastic t-test.

From previous studies it has been shown that in addition to making the membrane permeable, curcumin appears to induce an apoptosis-like response involving RecA in *E*. *coli* [4]. As a first step to understanding how this response relates to and interacts with the MscL-dependent permeabilization, we compared the response to curcumin treatment of *E*. *coli* WT and RecA null strains with or without MscL expression. As shown in Fig 4, both RecA and MscL are required for growth inhibition (Fig 4A) and decrease in viability (Fig 4B) upon curcumin treatment.

### Potassium and Glutamate *in vivo* flux experiments indicate that MscL is activated by curcumin independent of RecA expression

**Fig 5:**
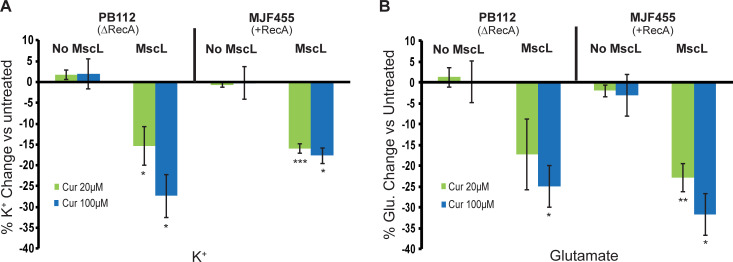
Curcumin induces a MscL-dependent reduction in bacterial K^+^ and glutamate steady state. Shown are the *E*. *coli* Stains PB112 (ΔRecA, ΔMscL, ΔMscS) and MJF455 (ΔMscL, ΔMscS) carrying vector only (No MscL) or expressing MscL (MscL), treated with curcumin at 20 μM (green) or 100 μM (blue). A) The reduction of potassium was measured after overnight treatment of curcumin (as indicated) and expressed as the percentage change of K^+^ remaining compared to untreated. B) The reduction of glutamate was similarly measured as the percent change vs Untreated. n = 3. Statistics reflect a comparison of MscL-expressing versus “no MscL” strains *P < 0.05, **P < 0.005, ***P < 0.0005 as indicated, 2-tailed, homoscedastic t-test.

Next, we determined if MscL activation by curcumin was RecA dependent or if these are two independent and complementary mechanisms. Both K^+^ and glutamate are known to flux out the bacterial cells upon MscL activation [15,18,22]. We therefore measured the flux of these ions in response to curcumin treatment in cells with and without RecA and MscL; the amount of these ions remaining in the bacterial cells after curcumin treatment was compared to untreated cells. As shown in Fig 5, both K^+^ (Fig 5A) and glutamate (Fig 5B) flux out of the bacterial cell upon curcumin treatment in a MscL-dependent manner, reflected as a decrease in the bacterial content of these ions after curcumin treatment. These effects are dependent on the curcumin concentration but independent of the RecA status of the bacterial strain. Collectively, these results imply that while both RecA and MscL are required for growth inhibition, MscL is activated by curcumin independent of the presence of RecA, but this activation alone is insufficient for significant decreases in growth (see Fig 4).

### Curcumin synergy with other antibiotics is MscL-dependent but RecA-independent, suggesting curcumin-dependent MscL opening allows cytoplasmic access of the antibiotics

**Fig 6:**
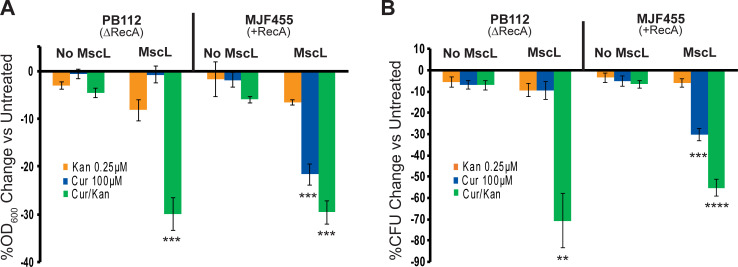
Curcumin antibiotic synergy is MscL-dependent but RecA-independent. Shown are the *E*. *coli* Stains PB112 (ΔrecA, ΔMscL, ΔMscS) and MJF455 (ΔMscL, ΔMscS) carrying vector only (No MscL) or expressing MscL (MscL), treated with 0.25 μM kanamycin (yellow), 100 μM curcumin (blue), or a combination of the two (green). A) Inhibition of growth (OD_600_) treated as indicated, expressed as the percentage change of the untreated. n = 4, Statistics reflect a comparison of MscL-expressing versus “no MscL” strains ***P < 0.0005 as indicated, 2-tailed t-test.

Curcumin has been shown to increase the potency of many drugs that require cytoplasmic access [7,8,9]. In addition, MscL agonists have also been shown to act as potentiators of known antibiotics by facilitating their access to the bacterial cell [14,15,16,17,18]. We therefore tested if the previously reported increased potency of known antibiotics by curcumin is due to MscL activation. To separate the effects of curcumin in cell growth and viability from the activation of MscL, we again used *E*. *coli* WT and null for RecA strains, with or without expression of MscL. We treated cells with a subthreshold concentration of the aminoglycoside Kanamycin alone, with curcumin alone, and with a combination of the two, and measured their effects on growth (Fig 6A) and viability (Fig 6B). As expected, the subthreshold concentration of Kanamycin had no effect independent of the presence of RecA or MscL, and treatment with curcumin alone had a detrimental effect on growth and viability if both RecA and MscL were present. Interestingly, Kanamycin and curcumin had additive or synergetic effects that were MscL-dependent, but independent of the RecA status of the strain. These results suggest that the potentiation of Kanamycin by curcumin is due to its activation of MscL. Furthermore, consistent with these findings, a potentiation of tetracycline effect by curcumin is also MscL-dependent; it was also similar to the observed effect of the previously described MscL agonist 011A [16,17] and the combination of the three (S1 Fig and S1 Table). A likely interpretation of these data is that like the other agonist 011A and K05, curcumin activates MscL, allowing a pathway to the cytoplasm for the other antibiotics.

### Inhibition of septation by curcumin is MscL-dependent

**Fig 7:**
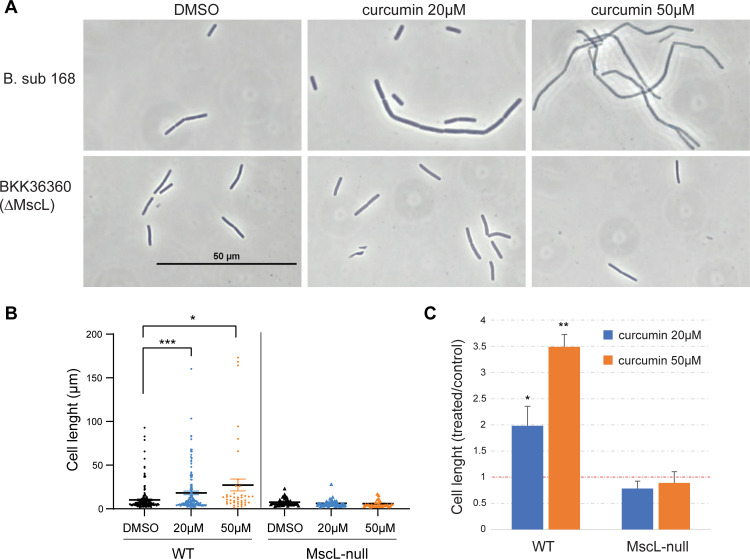
MscL is required for the inhibition of septation by curcumin. A. Shown are representative pictures of cultures of the *B sub* strain 168 (WT) and its MscL-null derivative, grown under different curcumin concentrations. B. The graph shows the length of the cells measured for both strains, under different treatments. ***p<0.005, *p<0.02 (Unpaired t test with Welch’s correction). C. A comparison of the change in cell length after curcumin treatment, presented as the ratio between treated and untreated cells, for WT and MscL-null strains. *p<0.05 **p<0.005 t-test, unpaired.

Curcumin treatment has been associated with the impairment of septation by inhibition of FtsZ assembly [6]. To assess the MscL dependency of this effect, we used *Bacillus subtilis* (B. sub), in which the *in vivo* filamentous cell formation was first observed; we compared the parental strain, Bacillus subtilis subsp. Subtilis 168, to its MscL-null derivative BKK36360 [23]. *B*. *subtilis*, WT and MscL-null cultures were treated for 90 minutes with different curcumin concentrations and the length of the cells measured. As shown in Fig 7A, the *B*. *subtilis* WT strain showed the formation of long filamentous cells in a curcumin-concentration-dependent manner; interestingly, filamentous cell formation was also MscL dependent. A quantification of this phenomenon is presented in Fig 7B, in which the length of the cells is compared, shows that some cells undergo this snaking process in the WT strain, but it is clearly absent in the MscL-null strain. A comparison of the average cell length increase after curcumin treatment is shown in Fig 7C, where a clear increase in cell length is seen for the WT group in a curcumin-concentration-dependent manner but remains unchanged in the MscL-null strain. Although statistically, there is no significant difference between the DMSO control cells with and without MscL, there is a small population of cells that are filamentous and are largely seen in the cells expressing MscL, as illustrated in Fig 7. The all points histogram in Fig 7B clearly shows these outliers, with cells longer than 20μm accounting for 10% of cells expressing MscL, but only 2% of those not expressing MscL. Similar results were also seen in cultures without DMSO (9% of cells in cultures expressing MscL, but only 4% of cells in cultures without MscL, were greater than 20μm in length, and 3% of cells in cultures expressing MscL but 0% of cells in cultures not expressing MscL were greater than 65μm). Together, the data suggest MscL plays a normal role in inhibiting septation in some cells, and show that MscL activation by curcumin leads to pathologically significantly increases in these inhibitory effects on septation.

## Discussion

Previous studies have shown that for curcumin’s antibacterial activity there are at least three modes of action: an apoptosis-like response involving RecA [4], membrane permeabilization [5], and inhibition of septation involving FtsZ [6]. Curcumin has also been shown to work synergistically with other antibiotics [7,8,9]. Here we find that in *E*. *coli* both MscL and RecA are required for curcumin-dependent decreases in growth and viability; in addition, curcumin’s membrane permeabilization, inhibition of septation in *B*. *subtilis*, and ability to work in synergy with other antibiotics are dependent upon the MscL channel.

Curcumin clearly activates MscL, as assayed by *in vivo* physiology and flux studies, as well as by patch clamp studies in native bacterial membranes. Given that curcumin intercalates in the membrane, one might expect that it influences multiple membrane proteins; however, using controls such as MscL-null cells and MscS overexpression we found exquisite specificity for the MscL channel in such things as influences on growth, viability and synergy with other antibiotics. But our studies do not address if or where curcumin directly binds the MscL channel, or if it somehow modulates MscL gating by modifying the biophysical properties of the membrane in a way that MscL is hyper-sensitive to. Preliminary experiments suggest that curcumin does not bind in the two known binding pockets: in the pore [15], or along the cytoplasmic surface of the protein [16,17,18] (see S2 Fig). In addition, there are indications that the different lipid compositions of mammalian, yeast and bacterial membranes determine curcumin distribution within the lipid bilayer and thus the alteration of membrane properties [24], which could conceivably partially explain bacterial-membrane specificity for altering membrane-protein function. Curcumin **is** also thought to thin the membrane [25]; and there are indications that MscL may be somewhat more sensitive to membrane thinning than our control channel, MscS [26]. On the other hand, every other compound that we know of that shows MscL specificity, with no MscS-dependency, does directly bind MscL, and all compounds we know of that perturb the membrane affect both MscL and MscS. In addition, one study looked at the effects of five phytochemicals, including curcumin, on the activity of four different membrane proteins, including MscL [27]; unlike the other proteins studied, MscL was suspiciously 300-fold more sensitive to curcumin than the other phytochemicals tested, consistent with a direct interaction. Hence, curcumin is either the first compound to show MscL specificity without direct binding, or, perhaps more likely, curcumin directly binds MscL at a site that has yet to be determined; distinguishing these possibilities is a matter for future study.

A likely mechanism for the influence of MscL on *B*. *subtilis* FtsZ polymerization and cellular septation is ion flux upon MscL gating. One study showed that not only is filamentous cell formation in *B*, *subtilis* observed upon curcumin treatment, but also potassium and phosphorus appear to flux from the cytoplasm [28], which would be consistent with MscL activation. Divalent ions have been shown to modulate FtsZ assembly [29], and MscL-dependent Ca^++^ fluxes have been observed *in vitro* [30] as well as *in vivo* from a Synechocystis bacterial strain [31]. In Fig 7, there is also an obvious outlier population of filamentous cells observed in MscL-containing untreated cultures that is mysteriously absent in cells null for MscL; thus, the data suggest that MscL may play a normal physiological role in modulating septation; this regulation is amplified upon curcumin treatment but disrupted upon deleting MscL.

Recent studies have shown that MscL agonists open the large MscL pore, thus allowing solutes out of, and drugs into the bacterial cell [16,17,18]. Curcumin clearly leads to channel gating: as with other MscL activators [10,14,15,16,17,18,32,33], curcumin increases the probability of MscL channel opening in patch clamp (**Fig 3**). In this light, the experimental data for curcumin increasing membrane permeability, both past and present, are thus entirely expected. In previous studies, curcumin has been shown to make the cell permeable to both propidium iodide and calcein, the latter of which has a hydrodynamic radii of 7.4 Å and has been routinely used as an assay for MscL gating in potential drug-delivery devices [10,34,35]. Here we show that curcumin activation leads to MscL-dependent leakage of the osmoprotectants potassium and glutamate, as has been seen previously with MscL activation [10,16,17,18]. Previous studies have shown that MscL agonists potentiate other antibiotics, or even their own activity, either by facilitating access into the cytoplasm or potentiating MscL activation. The list includes ampicillin, which weakens the cell wall, increases the tension in the cytoplasmic membrane, and thus also increases the probability of MscL opening. Ampicillin therefore shows potentiation with MscL agonists—the duo leads to even more detrimental MscL activity than either alone [16]. In addition, we have previously shown that streptomycin binds to, activates, and thus uses MscL as a pathway into the cell [15]. The most common mechanism of potentiation studied, however, is potentiation by allowing other antibiotics access to the cytoplasm through the open MscL channel [14,15,16,17,18,33]. Curcumin has also been previously shown to work synergistically with other antibiotics [7,8,9], and here we show this synergy is MscL-expression-dependent, as demonstrated with kanamycin and tetracycline. Perhaps most convincingly, in ΔRecA cells, at a concentration where curcumin has little measurable effect on the growth or viability of the cells on its own, curcumin still serves as an adjuvant increasing the potency of other antibiotics in a MscL-dependent manner (see **Fig 6**).

Given these findings, it seems likely that the further study of curcumin-like compounds and other modulators of MscL activity could lead to an era of MscL-activating adjuvants that would allow lower concentrations of antibiotics to be used for treatment; this could decrease potential side effects, allow shunting of multidrug resistant efflux pumps, and overall be critical for the treatment of drug resistant infections.

## Methods

### Strains and cell growth

The pB10d expression vector was used alone or with constructs inserted for expression; note that this is a mid-expression vector and expresses MscL at only a few times endogenous levels [21]. The E. *coli* Frag 1 derived cell lines, MJF367 (ΔmscL::Cam), MJF451 (ΔMscS), MJF455 (ΔmscL::Cam, ΔMscS), [36] PB112, which is MJF455 made ΔrecA (ΔrecA mscL::Cam, ΔMscS) as described [37], MJF612 (ΔmscL::cm, ΔmscS, ΔmscK::kan, ΔybdG::aprΔ) [38] were used as a hosts. Cultures inoculated from a single colony were grown either in citrate-phosphate-defined media (CphM) pH 7.0, consisting of per liter: 8.57 g of Na_2_HPO_4_, 0.87 g of K_2_HPO_4_, 1.34 g of citric acid, 1.0 g NH_4_SO_4_, 0.001 g of thiamine, 0.1 g of MgSO47H2O, 0.002 g of (NH_4_)2SO_4_.FeSO_4_.H_2_O, or K10 media containing 46 mM Na_2_HPO_4_, 23 mM NaH_2_PO_4_, 8 mM (NH_4_)_2_SO_4_, 10 mM KCl, and 100 mM NaCl, supplemented with 0.4 mM MgSO_4_, 3 μM thiamine, 6 μM iron, 0.04% glucose, ampicillin 100 μg/ml and incubated in a 37°C shaker, rotated at 250 cycles per minute.

### *In Vivo* assays

#### Growth experiments

Growth inhibition was measured as previously described [17]. Briefly, overnight cultures of MJF376, MJF451, MJF455, PB112 or MJF612 strains carrying the indicated constructs, were diluted 1:50 in CphM and grown until an OD_600_ of 0.2 was reached. Expression was then induced by the addition of 1 mM isopropyl-b-D-thiogalactopyranoside (IPTG) for 30 minutes, 10 mM stocks of curcumin (Sigma,-Aldrich, St. Louis, MO) solubilized in sterile dimethyl sulfoxide (DMSO) (Sigma,-Aldrich, St. Louis, MO), was diluted to two times its final concentration in pre-warmed CphM with a final DMSO concentration of 2% and 100 μl was added to wells of a pre-warmed, sterile 96 well flat bottom plate (Greiner bio-one, Monroe, NC). Cultures were then diluted 1:200 in pre-warmed CphM, 100 μg/ml ampicillin and 2 mM IPTG with diluted experimental antibiotics, were indicated, at 2X their concentration or mock (DMSO only). Final concentrations of antibiotics were: Tetracycline Hydrochloride 0.2 μM (Thermo Fisher Scientific Waltham, MA) 011A 40 μM [17] (3-(2-Chlorophenyl)-5-methylisoxazole-4-carboxylic acid, Sigma,-Aldrich, St. Louis, MO), kanamycin A at 0.25 μM (Sigma Aldrich St. Louis, MO, CA), 100 μl of culture mixture was added to the 96 will plates for a total of 200 μl, sealed with a sterile breathable film (Axygen, Union City, CA), wrapped in aluminum foil and placed in a 37°C shaker, rotated at 110 Cycles per minute for 16–17 hours and OD_620_ was then taken with a Multiskan Ascent 354 (Thermo Fisher Scientific Waltham, MA) plate reader with appropriate blank controls.

#### Viability experiments

Cultures from the above overnight growth experiments were used for all viability experiments done as previously described [16,17,18]. Briefly, without adjusting for OD, cultures were diluted 1:20 into pre-warmed CphM, serially diluted from 10^3^ to 10^6^ in a 96 well plate and liquid drops of 5 μl were placed on a pre-warmed LB ampicillin plates and placed in a 37°C incubator. The next morning colony-forming units were calculated to determine cell viability, as previously described [39,40].

#### Electrophysiology

*E*. *coli* giant spheroplasts were generated from MJF612 strain expressing Eco-MscL as previously described [21]. Patch clamp experiments were performed in excised patches in the inside-out configuration, at room temperature under symmetrical conditions; the buffer consisting of 200mM KCl, 90mM MgCl2, 10mM CaCl2 and 5mM HEPES adjusted to pH 7.0. Measurements were made for each patch before (control) and after the addition on a 100μM curcumin solution in a final 2% DMSO. Recordings were performed at 20mV (for simplicity the patch traces openings are shown upward) and data acquired at a sampling rate of 20 kHz with 10 kHz filtration using an AxoPatch 200B amplifier (Molecular Devices, Sunnyvale, CA, USA). A piezoelectric pressure transducer (World Precision Instruments, Sarasota, FL, USA) was used to measure the pressure applied to the patch throughout the experiments.

#### Steady state K^+^

The PB112 and MJF455 strains were used for the K^+^ steady state experiments carrying either pb10d empty vector or expressing WT *Eco-*MscL in the same pb10d vector. As previously described (Wray 2020), overnight cultures were grown in K10 media from a single colony, diluted 1:25 in the same media, grown to an OD_600_ of 0.2, then expression was induced with 1 mM IPTG for 1 hour for a final OD_600_ of about 0.4. Cultures were then split and treated with: curcumin at 20 μM, curcumin 100 μM or mock (DMSO only), with a final DMSO concentration of 2%. Cultures were then grown for 16 hours in a 37°C shaker. The final OD_600_ was then recorded and the volume of culture to be used was adjusted from the OD value. Cultures were passed through a 0.45-μm filter, washed with K0 media (K10 with the KCL replaced by NaCl) without ampicillin or supplements. The filters were then placed in plastic beakers, covered with foil and dried in an 80°C oven overnight. The next morning filters were rehydrated in 3 ml of double distilled water and K^+^ was measured using a Jenway flame photometer (Cole-Palmer, East Norwalk, CT).

#### Glutamate steady state

Samples from the above K^+^ steady state experiments (4 ml) were pelleted and stored at -80°C for later glutamate testing. Pellets were brought up in K10 media adjusting volume for OD and sonicated for 2 min. Glutamate measurements were preformed using a EnzyChrom Glutamate Asssay Kit (BioAssay Systems, Hayward, CA).

#### Cell length quantification

Bacillus subtillis strains 168 and BKK36360 [23] were purchased from the Bacillus Genetic Stock Center (Columbus, OH). Overnight cultures were grown in LB, diluted next day to 1:100, grown two hours and then diluted again and split in three groups treated with, DMSO 2% (control), curcumin 20 or 50 μM. After 90 minutes cells were fixed under microscope slides and pictures were taken. The length of the cells was measured using ImageJ software.

## Supporting information

S1 Fig Curcumin can increase the potency of other antibiotic compounds. Shown is the *E*. *coli* strain MJF612 (ΔMscL, ΔMscS, ΔMscK, ΔybdG) carrying vector only (no MscL) or expressing MscL (red), treated with (i) curcumin 100 μM (ii) tetracycline 0.2 μm (iii) compound 011A 40 μM (iiii) or a combination of two or all three as indicated. A) Inhibition of growth (OD_600_) expressed as the percentage change vs untreated culture. n = 3 B) The reduction in viability of cultures shown in A, expressed as the percent change of colony forming units (CFUs) vs Untreated. n = 3 Note a 50% decrease in growth and a 90% decrease in viability when curcumin is used in combination with 011A and tetracycline.
(PDF)

S2 Fig Curcumin does not bind to know MscL binding sites. Shown is the *E*. *coli* Stains MJF455 (ΔMscL, ΔMscS) carrying either vector only (no MscL) or MscL constructs as indicated. A) Inhibition of growth (OD_600_) of MscL constructs known to be involved in 011A binding, treated with curcumin at 100 μM (blue) or 175 μM (red) and expressed as the percentage change vs untreated culture are shown. Note that unlike compounds 011A and K05 treated constructs, *B*. *sub* WT and *E*. *coli* K97R show the same reduction in growth as the *E*. *coli* WT construct. n = 3, **P < .005, as indicated, 2-tailed, homoscedastic T test. B) The reduction in viability of cultures shown in A, expressed as the percent change of colony forming units (CFUs) vs Untreated. n = 3, *P < .05, **P < .005 as indicated, 2 tailed, homoscedastic T test. C) Inhibition of growth (OD_600_) of constructs known to be involved in dihydrostreptomycin binding, treated with curcumin at 100 μM (grey) or 175 μM (green) and expressed as the percentage change vs untreated culture are shown. Note that no significant difference in growth was seen for *E*. *coli* L19M or *H*. *inf* WT that were observed with dihydrostreptomycin. n = 3, *P < 0.05, **P < 0.005 as indicated, 2 tailed, homoscedastic t-test. D) The reduction in viability of cultures shown in C, expressed as the percent change of colony forming units (CFUs) vs Untreated. n = 3, *P < 0.05, **P < .005, 2 tailed, homoscedastic t-test.
(PDF)

S1 Table Shows the statistics for S1 Fig (p-values) for a 2-tailed, unpaired homoscedastic T-test. The significant difference between curcumin+tetracycline+011A and 011A alone in the viability appears to be because the Tet samples yielded a decrease in viability not seen with 011A (the latter is because of the absence of MscL). We thus show the significance of all of the viability no MscL samples containing Tet against 011A alone to demonstrate this point.
(PDF)
