# Supplementary material for: Curcumin activation of a bacterial mechanosensitive channel underlies its membrane permeability and adjuvant properties
Source: PLoS Pathog. 2021 Dec 23;17(12):e1010198. doi: 10.1371/journal.ppat.1010198 (PMC8769312; doi:10.1371/journal.ppat.1010198)
Supplement: S1 Fig — Shown is the E. coli strain MJF612 (ΔMscL, ΔMscS, ΔMscK, ΔybdG) carrying vector only (no MscL) or expressing MscL (red), treated with (i) curcumin 100 μM (ii) tetracycline 0.2 μm (iii) compound 011A 40 μM (iiii) or a combination of two or all three as indicated. A) Inhibition of growth (OD600) expressed as the percentage change vs untreated culture. n = 3 B) The reduction in viability of cultures shown in A, expressed as the percent change of colony forming units (CFUs) vs Untreated. n = 3 Note a 50% decrease in growth and a 90% decrease in viability when curcumin is used in combination with 011A and tetracycline. (PDF) [file ppat.1010198.s001.pdf]

Supplemental Figure 1

We assayed Curcumin to work synergistically with tetracycline (Tet). As seen in Figure S1, below, curcumin increased the potency of Tet in a manner similar to the MscL agonist compound 001A [1, 2].

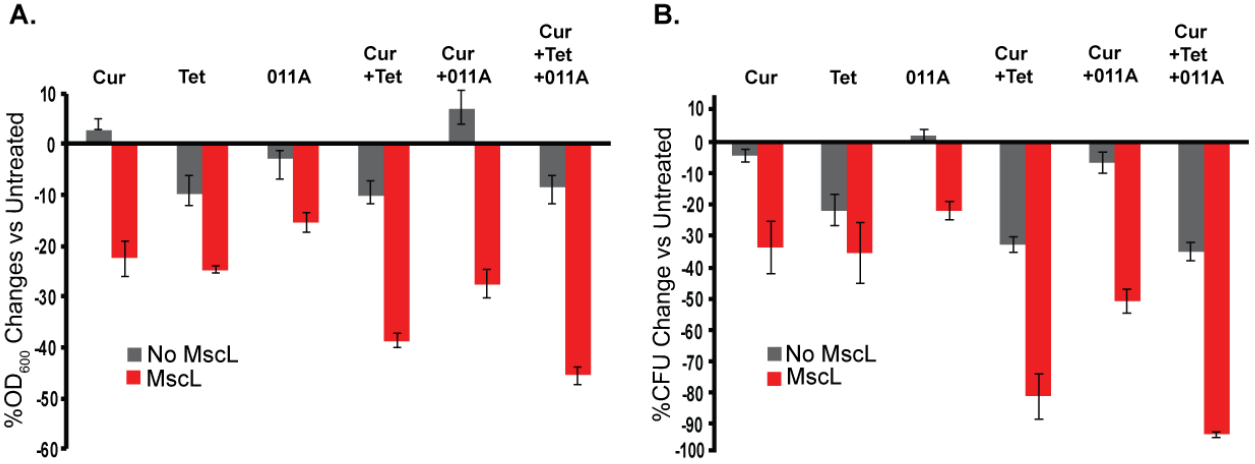

S1. <u>Curcumin can increase the potency of other antibiotic compounds.</u> Shown is the *E. coli* strain MJF612 (ΔMscL, ΔMscS, ΔMscK, ΔybdG) carrying vector only (no MscL) or expressing MscL (red), treated with (i) curcumin 100 µM (ii) tetracycline 0.2 µm (iii) compound 011A 40 µM (iiii) or a combination of two or all three as indicated. A) Inhibition of growth (OD_{600}) expressed as the percentage change vs untreated culture. n=3 B) The reduction in viability of cultures shown in A, expressed as the percent change of colony forming units (CFUs) vs Untreated. n=3 Note a 50% decrease in growth and a 90% decrease in viability when curcumin is used in combination with 011A and Tet.

1. Wray R, Herrera N, Iscla I, Wang J, Blount P. An agonist of the MscL channel affects multiple bacterial species and increases membrane permeability and potency of common antibiotics. *Mol Microbiol* **112**, 896-905 (2019).

2. Wray R, Iscla I, Kovacs Z, Wang J, Blount P. Novel compounds that specifically bind and modulate MscL: insights into channel gating mechanisms. *Faseb J* **33**, 3180-3189 (2019).
