# Supplementary material for: Curcumin activation of a bacterial mechanosensitive channel underlies its membrane permeability and adjuvant properties
Source: PLoS Pathog. 2021 Dec 23;17(12):e1010198. doi: 10.1371/journal.ppat.1010198 (PMC8769312; doi:10.1371/journal.ppat.1010198)
Supplement: S2 Fig — Shown is the E. coli Stains MJF455 (ΔMscL, ΔMscS) carrying either vector only (no MscL) or MscL constructs as indicated. A) Inhibition of growth (OD600) of MscL constructs known to be involved in 011A binding, treated with curcumin at 100 μM (blue) or 175 μM (red) and expressed as the percentage change vs untreated culture are shown. Note that unlike compounds 011A and K05 treated constructs, B. sub WT and E. coli K97R show the same reduction in growth as the E. coli WT construct. n = 3, **P < .005, as indicated, 2-tailed, homoscedastic T test. B) The reduction in viability of cultures shown in A, expressed as the percent change of colony forming units (CFUs) vs Untreated. n = 3, *P < .05, **P < .005 as indicated, 2 tailed, homoscedastic T test. C) Inhibition of growth (OD600) of constructs known to be involved in dihydrostreptomycin binding, treated with curcumin at 100 μM (grey) or 175 μM (green) and expressed as the percentage change vs untreated culture are shown. Note that no significant difference in growth was seen for E. coli L19M or H. inf WT that were observed with dihydrostreptomycin. n = 3, *P < 0.05, **P < 0.005 as indicated, 2 tailed, homoscedastic t-test. D) The reduction in viability of cultures shown in C, expressed as the percent change of colony forming units (CFUs) vs Untreated. n = 3, *P < 0.05, **P < .005, 2 tailed, homoscedastic t-test. (PDF) [file ppat.1010198.s002.pdf]

Supplemental Figure 2

In previous studies we identified the binding site on MscL for the novel compounds 011A [1,2] and K05 [3] as well as for dihydrostreptomycin (DHS) [4].

For 011A [1] and K05 [3], the binding pocket in the MscL channel, along the cytoplasmic interface, is not conserved in the *B. subtilis* orthologue; there is a K to R substitution at one of the sites within the pocket. As a consequence, cells expressing the *B. subtilis* channel are unresponsive to the 011A and K05 compounds. Consistent with this finding, cells expressing the *E. coli* channel with a K to R substitution at this position did not respond to 011A or K05. However, cells expressing the *B. subtilis MscL* where a mutation at this site was made from R to K, making the site to the canonical binding site, *were* now sensitive to the 011A and K05 compounds. As seen in Figure S2, for the compound curcumin we saw neither a difference in efficacy for cells expressing *B. subtilis* MscL nor the mutants mentioned above in either growth (Panel A) or viability (Panel B).

Similarly, for DHS we previously noted that *H. influenzae* lacked the canonical binding site in the pore, having an M rather than L at position 19, and mutation of the site influenced efficacy of DHS, either in a negative (*E. coli* L19M) or positive *H. influenzae* M19L) way [4]. However, here again found that curcumin had no difference in efficacy for cells expressing the *H. influenzae* MscL or any of the mutants mentioned above in either growth (Panel C) or viability (Panel D).

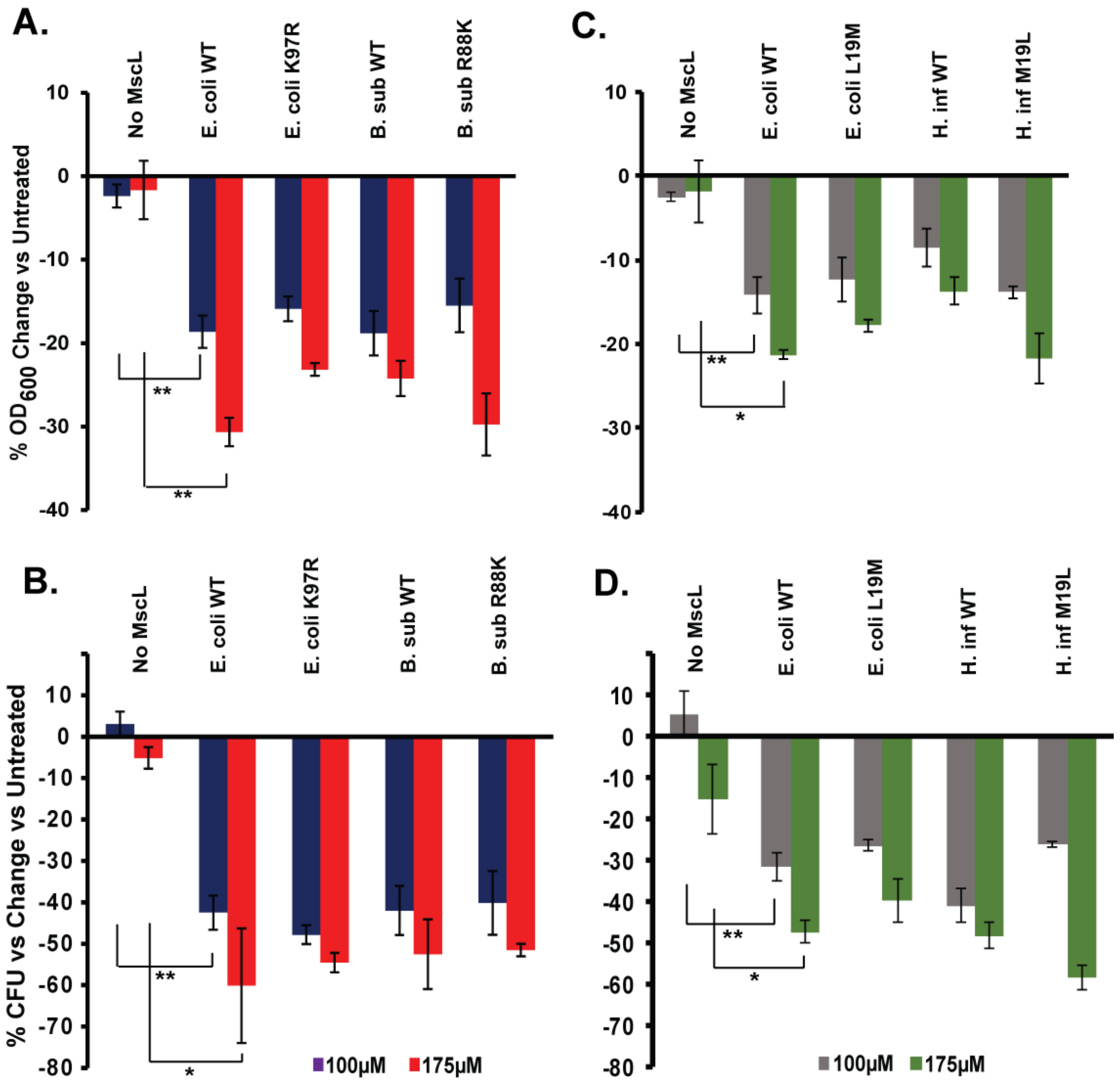

S2<u>. Curcumin does not bind to know MscL binding sites.</u> Shown is the *E. coli* Stains MJF455 (ΔMscL, ΔMscS) carrying either vector only (no MscL) or MscL constructs as indicated. A) Inhibition of growth (OD_{600}) of MscL constructs known to be involved in 011A binding, treated with curcumin at 100 μM (blue) or 175 μM (red) and expressed as the percentage change vs untreated culture are shown. Note that unlike compounds 011A and K05 treated constructs, *B. sub* WT and *E. coli* K97R show the same reduction in growth as the *E. coli* WT construct. n=3, **P<.005, as indicated, 2-tailed, homoscedastic T test. B) The reduction in viability of cultures shown in A, expressed as the percent change of colony forming units (CFUs) vs Untreated. n=3, *P<.05, **P<.005 as indicated, 2 tailed, homoscedastic T test. C) Inhibition of growth (OD_{600}) of constructs known to be involved in dihydrostreptomycin binding, treated with curcumin at 100 μM (grey) or 175 μM (green) and expressed as the percentage change vs untreated culture are shown. Note that no significant difference in growth was seen for *E. coli* L19M or *H. inf* WT that were observed in dihydrostreptomycin binding. n= 3, *P<.05, **P<.005 as indicated, 2 tailed, homoscedastic T test. D) The reduction in viability of cultures shown in C, expressed as the percent change of colony forming units (CFUs) vs Untreated. n=3, *P<.05, **P<.005, 2 tailed, homoscedastic T test.

References:

1. Wray R, Iscla I, Kovacs Z, Wang J, Blount P (2019) Novel compounds that specifically bind and modulate MscL: insights into channel gating mechanisms. Faseb J 33: 3180-3189.
2. Wray R, Herrera N, Iscla I, Wang J, Blount P (2019) An agonist of the MscL channel affects multiple bacterial species and increases membrane permeability and potency of common antibiotics. Mol Microbiol 112: 896-905.
3. Wray R, Wang J, Iscla I, Blount P (2020) Novel MscL agonists that allow multiple antibiotics cytoplasmic access activate the channel through a common binding site. PLoS One 15.
4. Wray R, Iscla I, Gao Y, Li H, Wang J, et al. (2016) Dihydrostreptomycin Directly Binds to, Modulates, and Passes through the MscL Channel Pore. PLoS Biol 14: e1002473.
