# Supplementary material for: Curcumin activation of a bacterial mechanosensitive channel underlies its membrane permeability and adjuvant properties
Source: PLoS Pathog. 2021 Dec 23;17(12):e1010198. doi: 10.1371/journal.ppat.1010198 (PMC8769312; doi:10.1371/journal.ppat.1010198)
Supplement: S1 Table — The significant difference between curcumin+tetracycline+011A and 011A alone in the viability appears to be because the Tet samples yielded a decrease in viability not seen with 011A (the latter is because of the absence of MscL). We thus show the significance of all of the viability no MscL samples containing Tet against 011A alone to demonstrate this point. (PDF) [file ppat.1010198.s003.pdf]

Supplemental Table S1

Table S1 shows the statistics for Figure S1 (p-values) for a 2-tailed, unpaired homoscedastic T-test. The significant difference between Curcumin+TET+011A and 011A alone in the viability appears to be because the Tet samples yielded a decrease in viability not seen with 011A (the latter is because of the absence of MscL). We thus show the significance of all of the viability no MscL samples containing Tet against 011A alone to demonstrate this point.

Growth

MscL

	Vs TET ONLY	Vs 011A only
Cur + TET	0.0010	
Curcumin+011A		0.0265
Curcumin+TET+011A	0.0004	0.0004

No MscL

	Vs TET ONLY	Vs 011A only
Cur + TET	0.9367	
Curcumin+011A		0.0604
Curcumin+TET+011A	0.7326	0.1058

Viability

MscL

	Vs TET ONLY	Vs 011A only
Cur + TET	0.01987	
Curcumin+011A		0.00391
Curcumin+TET+011A	0.00405	0.00002

No MscL

	Vs TET ONLY	Vs 011A only
Cur + TET	0.1201	
Curcumin+011A		0.1006
Curcumin+TET+011A	0.0778	0.0005
Curcumin+TET		0.0004412
TET		0.0113
